# LONG-TERM PANDEMIC EFFECTS ON SOCIAL ACTIVITY AND LONELINESS AMONG OLDER ADULTS IN AN URBAN COMMUNITY

**DOI:** 10.1093/geroni/igae098.2334

**Published:** 2024-12-31

**Authors:** Rebecca Lindsay, Angela Gaye, Meosia Lee-Turner, Yolanda Hill-Ashford, Robin Brewer, Sheria Robinson-Lane, Mary Janevic

**Affiliations:** University of Michigan, Ann Arbor, Michigan, United States; Detroit Health Department, Detroit, Michigan, United States; Detroit Health Department, Detroit, Michigan, United States; Detroit Health Department, Detroit, Michigan, United States; University of Michigan, Ann Arbor, Michigan, United States; University of Michigan, Ann Arbor, Michigan, United States; University of Michigan, Ann Arbor, Michigan, United States

## Abstract

The COVID-19 pandemic sharply curtailed social activity among older adults. In Detroit, an underserved area impacted by structural inequities, older adults were at particularly high risk for social isolation, given the digital divide and prolonged closures of senior and community centers. While daily life has now mostly normalized, little is known about the extent to which older adults have re-engaged in social activities following the lengthy disruption. We examined baseline data collected in 2023-24 from an ongoing randomized controlled trial in Detroit of the RESET intervention (Re-Engaging in Self-care, Enjoying Today). Participants rated their level of 1) loneliness and 2) social activity compared to pre-pandemic (1=much lower to 5=much higher). The sample (N=213) was 91% female and 85% African American, mean age 68 years (range 50 to 90), and 54% lived alone. Almost half (46.0%%) of the sample reported that their current social activity level was much lower or lower than pre-pandemic, and 34.8% that their loneliness level was much higher or higher. No significant differences were found by age group (under 65 vs 65+) or living alone vs. with others. We then examined open-ended responses to a question about continued pandemic impact. Major themes were: ongoing fear of COVID-19 infection, caution about gatherings/crowds, and persistent mood problems. These data show that for some older adults, the pandemic continues to have adverse effects on social activity and loneliness. Targeted interventions that address and accommodate older adults’ ongoing COVID-19 concerns may be needed to boost social functioning in this group.

